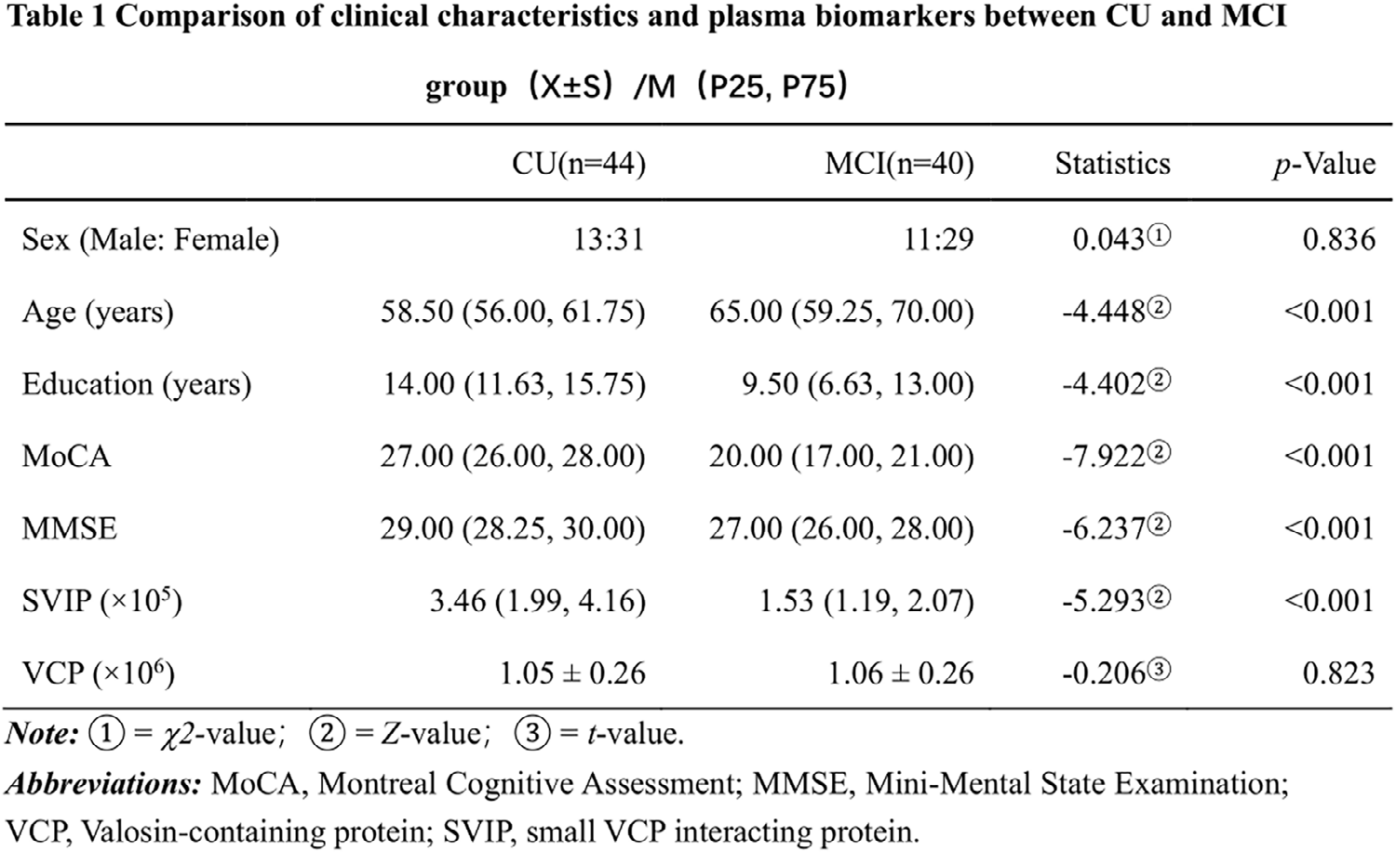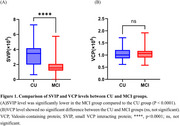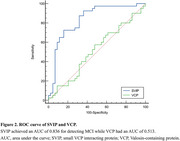# SVIP: A Potential Blood‐based Biomarker for Early detection of Mild Cognitive Impairment

**DOI:** 10.1002/alz70856_102799

**Published:** 2025-12-25

**Authors:** Gaigai Lu, Xiang Fan, Keyan Yu, Lele Chen, Zhuonan Wei, Hui Chen, Lin Hu, Guanxun Cheng, Hui Shan

**Affiliations:** ^1^ Peking University Shenzhen Hospital, Shenzhen, Guangdong, China

## Abstract

**Background:**

Mild Cognitive Impairment (MCI) represents a critical window for early detection of Alzheimer's Disease (AD). Although AD blood‐based biomarkers have developed rapidly in recent years, the concentrations of existing biomarkers, such as *p*‐tau (molecular weight: 50∼75 kDa) are generally low in the blood. Studies have demonstrated that increased expression of Valosin‐Containing Protein (VCP) can significantly enhance autophagy activity and reduce the level of phosphorylated and aggregated tau protein levels in animal models. Moreover, in the brain of AD patients, the expression of VCP is significantly downregulated and negatively correlated with *p*‐tau level. However, the diagnostic performance of VCP in peripheral blood has not been reported yet. Notably, VCP typically forms as a hexamer with a large molecular weight of 582 kDa, which may limit its ability to cross the blood‐brain barrier (BBB) even when the BBB breakdown happens. In contrast, the small VCP interacting protein (SVIP), with a molecular weight of 9 kDa, interacts with VCP to maintain the dynamic stability of autophagosomes within cells. Due to its much smaller size, we hypothesized that SVIP level in peripheral blood could be changed in MCI and aimed to evaluate whether plasma SVIP can outperform VCP in detecting MCI.

**Method:**

Eighty‐four participants, including 44 cognitively unimpaired (CU) individuals and 40 with MCI, were selected from the Shenzhen mulTi‐modal Aging Research (STAR) cohort. Plasma levels of SVIP and VCP were measured using deep plasma proteomics enriched with biofunctional magnetic beads. Statistical analyses were conducted using SPSS 25.0. Receiver operating characteristic (ROC) analysis was employed to evaluate performance.

**Result:**

Compared to the CU group, the MCI group showed significantly decreased SVIP (Z = ‐5.293, *p* < 0.001), while no significant difference was observed in VCP levels (t = ‐0.224, *p* = 0.823). The area under the curve (AUC) of SVIP in detecting MCI was 0.836 *(95%CI:0.739∼0.908)*while AUC of VCP was 0.513*(95%CI:0.401∼0.624)*.

**Conclusion:**

SVIP is a potential blood‐based biomarker for the detection of MCI, while the VCP Is unlikely to serve as a blood‐based biomarker, possibly due to its large hexameric forms.